# Gambling as Work: A Study of German Poker Players

**DOI:** 10.1007/s10899-023-10277-0

**Published:** 2023-12-22

**Authors:** Linus Weidner

**Affiliations:** 1 Department of Human and Social Sciences, University of Wuppertal, Gaußstr. 20, 42119 Wuppertal, Germany; 2 Institute for Gambling and Society (GLÜG), Ruhr-Universität Bochum, Bochum, Germany

**Keywords:** Gambling, Occupation, Poker, Serious leisure

## Abstract

The study addresses the question whether professional gamblers can be considered an occupational group from a sociological perspective. It combines survey data on poker players from the German state North Rhine-Westphalia with sociological theory in order to explain the oxymoron of professional gambling. The descriptive analysis of the survey data is supplemented by ego-centric network data of the poker players to analyze whether hobby and professional players maintain different forms of social relationships. Even when semiprofessionals and professional players are grouped together for the purpose of comparative analysis, they fulfill the criteria of occupational groups according to Salaman’s major work on the topic. The study points to the fact that more research on occupational groups outside of the common fields is needed and bridges the gap between the literature on serious leisure and sociological research on professions and professionalization.

## Introduction

The present article addresses the question whether (semi-)professional poker players form their own occupational community according to a sociological framework from Salaman (1974). When the average working hours declined along with the increased industrialization after World War I, researchers wondered whether people will put these additional hours of free time to good use or start “drinking or gambling” instead (van der Poel, 2006, p. 98). In modern times, the lines between “… ‘labour’ and ‘leisure’ have become internally differentiated and fragmented, the distinctions between these categories blurred or ‘de-differentiated’ (Rojek, 1995). There are now large grey areas between labour and leisure…” (van der Poel, 2006, p. 101). This might be exactly were (semi)professional gambling is located. Accordingly, the term ‘occupational community’ as is not introduced as a “classificatory heading” (Salaman, 1974, p. 127), but used as a tool to examine at the relationship between work and leisure from the perspective of (professional) poker players.

While this paper is not the first study to examine poker from a sociological perspective (Hayano, 1977, 1982, 1984; Istrate, 2011; McCormack & Griffiths, 2012) or to discuss occupational topics in relation to the gambling industry (Rosecrance, 1988; Sallaz, 2002), it is the first quantitative study on the topic using unique data of German players. This study is based on survey data of 109 German poker players ranging from amateurs to professionals. Researchers have mostly avoided (professional) poker players because they are clearly a hard to reach population. This also explains the relatively small sample in this study. Because of the small sample, the survey data is analyzed in a descriptive manner and linked to six criteria from Salaman’s framework. This study relates the survey results to Hayano’s (1977, 1982) major ethnographic work on poker players, enriching the descriptive analysis with accounts of historical development. The relevance of the social surroundings for different levels of poker players is considered via ego-centric network data that was collected alongside the survey data, relating the present study to the literature on the importance of social elements in the workplace (Volti, 2012, pp. 205–208) entrepreneurial networks (Fuentes et al., 2010; Singh et al., 1999; Vandekerckhove & Dentchev, 2005) and hobby networks (Arenius & Clercq, 2005).

It is important to study the characteristics of this group because professional gambling is clearly an under-researched topic from a work and occupation perspective and the people who work in the field might not get access to the normal protective mechanisms that other more recognized workers have. Instead, they might be a neglected community that is not supported by the government, the social security system or any employer associations. Moreover, occupational theory provides an idea about the societal position of professional gamblers more generally, since occupations are indicators of social position (Domański et al., 2009, pp. 21–42) or social class (Dunkerley, 1975, p. 37).

The article relates indirectly to the serious leisure (SLI) literature that studies the intensified engagement in a hobby (Stebbins, 1992) and to the casual leisure literature (Stebbins, 2001), which examines the hedonistic qualities of an activity, since not all participants in our study treat poker as a serious or somewhat professional activity, but purely as a hobby. Bergero (1962, p. 29) makes it clear, however, that the separation between (serious) leisure and work is primarily a normative question.

First, the article introduces relevant sociological literature on occupations. Next, it provides a short overview of the scientific literature on poker and on the few available empirical studies on gambling as an occupational activity. Afterwards, the process of data collection is explained and the findings from the survey are related to the hypotheses that are based on the six key criteria that Salaman identifies. On the basis of this analysis, it is concluded that (semi)professional poker players should be regarded as an occupational community. The final section discusses the main findings and limitations of the study and provides ideas for future research.

## Elements of an Occupational Activity

Sociologists tend to separate jobs into those that fulfill the very demanding criteria for professions (Abbott, 1988; Adams, 2010; Volti, 2012) and those that do not. It is clear that professional poker cannot be considered a true profession. Hayano (1982, p. 129) applied Ritzer’s (1972) classification of professionalism to professional poker players. He found that out of six aspects mentioned,^1^ only one—a distinctive culture—was fully applicable to professional poker. The criterion “community rather than self-interest” was not applicable at all since professional gambling is only pursued out of self-interest. However, Evetts (2003, p. 397) cautions that it is not useful to think of “true” professions and other occupational groups as two entirely different concepts, since they share many characteristics. Therefore, I analyze whether professional gamblers form an occupational community more generally, based on a framework from Salaman (1974). These communities “present a degree of convergence in work and non-work activities, interests and relationships….” (Salaman, 1974, p. 20). The term therefore allows a more flexible analysis of work/non-work relationships compared to other concepts (Volti, 2012, p. 156). Based on a synthesis and study of the available literature, Salaman combines three components of occupational groups and three determinants into a new model of occupational groups. The components are not regarded as necessary conditions or found in all occupations and they are strongly interconnected (Salaman, 1974, p. 27). Therefore, I use them as a guideline to get a first idea whether the most clearly identified components of occupational communities can be found in the (semi-)professional poker players. This is different for the determinants of occupational communities, which I examine afterwards. For the determinants, I would expect that (semi-)professional gamblers differ from hobby players. One of these determinants is mandatory (involvement) but one of the optional determinants also to has to be present to classify a group of workers as an occupational community.

Salaman makes one further important distinction. He only considers what he calls “true” occupational communities which form naturally (1974, p. 20f). Not included in his model are “quasi” occupational communities that exist because of local geographical characteristics or depend on a major monopolist company. However, he makes exceptions for occupational groups such as fisherman or coal miners and argues that—while they are geographically bound to a certain area—their occupational communities would exist regardless of it (Salaman, 1974, p. 21). (Semi-)professional gamblers also depend on the existence of casinos. Nevertheless, they might still form a cohesive community that is the basis for “association and identification” (Salaman, 1974, p. 20).

### Components of Occupational Communities

Salaman (1974, p. 21) claims that members of an occupational community self-identify as members of said group, view it as a reference group and seek insiders of the community as friends—either from the same workplace or more globally. Nevertheless, the identification can be problematic when people treat their jobs as “instrumental” (p. 22; 117). This might be the case if poker players are solely concerned about making money. I assume that (semi)professional poker players self-identify as such by attributing a lot of meaning to the activity, which is an important element for any kind of occupational work (Volti, 2012, p. 209). Similar to other occupations (Volti, 2012, pp. 205–208), meaning might be found in various social interactions that the players have with each other. Salaman (1974, p. 14f) argues that self-identification with an occupational community is important and shared values between the members of an occupation lead to a “reference group perspective”. The reference group seems to be especially important for deviant groups, to which the professional gambler might belong. One seemingly obvious argument related to the reference-group is that other people within the occupational community can be considered as colleagues (Salaman, 1974, p. 26). The networks of the players should therefore reveal this and will also be helpful in checking the last component of occupational communities, i.e. whether most (semi-)professional players establish poker-related friendships. From this discussion I derive the following hypotheses:

#### H1a

(Semi-)professional players self-identify as members of said community by treating the activity as an important aspect of their lives besides the financial aspects.

#### H1b

(Semi-)professional players regard other (semi-)professional players as a reference group by defining many of their poker-related social contacts as colleagues.

#### H1c

(Semi-)professional players consider other poker players as friends.

### The Three Determinants for Occupational Communities

Salaman (1974, p. 27) shows that three determinants are required for occupational communities to exist—involvement in work (see Gerstl, 1961), marginal status or stratification and an inclusiveness of the work or the organizational situation. Involvement is considered a mandatory determinant, but one of the other two conditions must be met as well for a community to qualify as “occupational” according to Salaman (1974, p. 37f). Involvement can be regarded as the opposite of alienation (Salaman, 1974, p. 119). He also explains that people tend to learn to value a work activity more the longer they are involved in it (Salaman, 1974, p. 29). This argument relates to psychological theories on positive self-attribution (Miller & Ross, 1975; Pronin et al., 2002) whereby people identify with tasks in order to avoid a negative self-image. Involvement is most likely an integral part of (semi-)professional play since it is a type of self-employed work and the player chooses his own way of working rather than fulfilling external tasks. Moreover, the required level of skill is one of the most important “involvement arousing factors” (Salaman, 1974, p. 120). For the (semi-)professional poker players, this involvement could be identified if they treat the activity as an important part of their life and not only care about the monetary aspects of it and if they estimate the required skill for their work higher than the hobby players.

Regarding the second determinant, Salaman writes: “In the context of this discussion an occupation can be described as marginal when members identify, and wish to associate, with members of a higher-status group and when these associational aspirations are unsuccessful” (Salaman, 1974, p. 30f). This is also called preferential-association (Lipset et al., 1956). Hayano (1984) considers professional poker players to be part of a marginal group that seeks defensive mechanism to deal with “outside-labeling and stereotyping” and according to Radburn and Horsley (2011, p. 30), professional poker players are situated at the edge between deviant and legitimate. However, “Status is only directly important as a determinant of occupational communities when there is a discrepancy between the status accorded an occupation by those involved in it and the status assessment of outsiders” (1974, p. 31). Unfortunately, the present study does not provide this outside perspective based on a representative survey of the German population and such data are not available. The marginal status will therefore probably be the most difficult part to test empirically. However, for a marginal status group I would expect a rather negative societal view of the group. If there is indeed an internal status hierarchy, hobby players should frequently strive to achieve professional status as well.

The third determinant for occupational communities is the *inclusiveness* of the work or organizational situation. This concept can be understood in three different ways. Firstly, there are varying levels of *organizational pervasiveness* (Etzioni, 1961, p. 163). The pervasiveness describes the norms set by the organization. This is clearly a minor factor in a poker setting, since there are only some basic house rules and table manners as well as the rules of the games. Most of the normative rules for accepted behavior are set by the players themselves, not by the organizations/providers, and there are not that many to speak of. Secondly, there is *organizational embrace* (Salaman, 1974, p. 34) or *organizational scope* as Etzioni (1961, p. 160) calls it. “Organizations which embrace their participants will attempt to ‘serve as the collectivity in which many or most of an individual’s activity will take place’” (Etzioni, 1961, p. 160 as cited in Salaman, 1974, p. 34). The *organizational embrace* is not a relevant factor for professional (semi-)professional poker players either. While the casinos offer some services to keep the players at the location, such as food and drinks, these are necessary conditions in order to be able to participate in any longer tournaments. Moreover, the pervasiveness and organizational embrace are limited simply due to the fact that the players do not work for the casinos or any organization. Finally, there are so called *restrictive factors* or “features of the job itself” (Salaman, 1974, p. 35) such as specific working hours or locations. However, because poker is usually played at nighttime and on weekends, I would expect that long working hours are a significant restrictive factor if (semi-)professionals play much more on average compared to the hobby players. Other studies also show that professional poker players play more frequently and for more hours per individual session (McCormack & Griffiths, 2012, p. 248). Similar to jazz musicians, this impacts the lifestyles and work-life balance of the (semi-)professional players in a significant way (Becker, 1963; Salaman, 1974, p. 35; 54f). This not only leads to an overlap between the private and the work life, which is also pronounced by the fact that many, if not all, (semi-)professionals pursued poker as a hobby at some point in their career, but also makes it increasingly difficult to maintain many social relationships outside of the industry. Moreover, the (semi-)professional players likely take much more personal risk by playing at higher stakes compared to hobby players and might not participate in other gambling activities because of the required specialization in poker. This leads to the following hypotheses:

#### H2a

(semi-)professional poker players have a high level of involvement in poker in comparison to hobby players.

#### H2b

(semi-)professional poker players are characterized by a marginal status or stratification situation.

#### H2c

(semi-)professional poker players experience an inclusiveness of the work or the organizational situation which is indicated by more limiting restrictive factors in comparison to hobby players.

## Empirical Studies on Professional Poker

Gambling has always been characterized by an element of luck. Diverse legislatory bodies have decided, that a game must contain a predominant proportion of luck to fall under gambling regulations. Games such as lotteries, scratch cards and slot machines are clearly games of pure luck and are therefore considered gambling. As they can only be beaten consistently by illegal fraud or manipulation, legal professional play is impossible. Some games such as poker contain an element of skill of varying proportion. Hence, it remains debatable whether skill or luck is more relevant for the long-term success in these games. For the game of poker, researchers have tried to examine whether skill or luck dominates (DeDonno & Detterman, 2008; Dreef et al., 2003; Fiedler & Rock, 2009; Kelly et al., 2007; Meyer et al., 2013). This is highly relevant in the context of this study, because people can only expect to make a steady income from the game and treat it as an occupational activity if the skill element dominates in the long term. Nevertheless, these studies arrive at contrary conclusions. I would argue that some researchers used insufficient playouts to get a statistically meaningful result (Meyer et al., 2013), while others argue only on a theoretical/regulatory basis (Kelly et al., 2007). Arguably the most promising study is from Fiedler and Rock (2009). They analyzed a survey population of 51,761 poker players. The authors conclude that poker is indeed a game of skill when time/playouts/populations are examined on a large scale. Siler (2010) also studied twenty-seven million hands from online poker players at all levels of skill/limits and describes the strategic approaches that are commonly chosen by players.

The available literature on occupational gambling is pretty limited. Rosecrance (1988) conducted a qualitative study of professional horse race gamblers, while Sallaz (2002) conducted an ethnographic study with dealers at blackjack tables in Las Vegas. The first of these two studies is therefore more closely related to the present article, since horse race gamblers work on their own and are not directly employed by an organization. Rosecrance (1988, p. 220) calls this the “bane and sustenance” of the occupation. McCormack and Griffiths (2012) also tried to explain how professional and recreational poker players differ from each other. They conducted a qualitative analysis with three professional, one semi-professional and five recreational players, identifying four important distinguishing factors for the professionals: treating poker as work, a logical and controlled approach to the game, minimizing risks and avoiding to chase losses. They find that “Playing poker for a living is very possible for a minority of players, but it takes a combination of talent, dedication, patience, discipline and disposition to succeed” (McCormack & Griffiths, 2012, p. 243). A related study was conducted by Zaman et al. (2014). They used laddering interviews to study the motivation of six professional, six semi-professional and six amateur online poker players in relation to the design characteristics of poker websites. Hopley et al. (2014, p. 14) also analyzed a sample of online poker players and found that workaholism in professional poker players is not identical to problem gambling. More recently, Newall and Talberg (2023) conducted qualitative interviews with 19 professional online poker players to explore the reasons for their success.

The life of the professional poker player has been researched before, usually from an ethnographic or qualitative perspective (Hayano, 1977, 1984; Holts, 2017; Holts & Surugiu, 2016; Radburn & Horsley, 2011). The study by Dufour et al. (2015) is an exception to this. They found via latent class analysis that internet-only players have the highest proportion of self-proclaimed experts and professionals (2015, p. 411). Therefore, I expect that internet play is more frequent in (semi-)professionals compared to hobby players. Hayano (1977, p. 558) identifies four types of professional poker players: The worker-pro who maintains another job alongside poker, the outside-supported professional, who has a different steady source of income such as pensions, welfare, savings, or working spouses/relatives, the subsistence-professional who usually plays the lower stakes and regards poker as a quick way to attain this subsistence, and the career-professional who is usually younger, unmarried or divorced and relies fully on his gambling income. Hayano also notices that these categories “do not necessarily represent stable, closed, occupational types” (Hayano, 1977, p. 558). This is in line with models based on the Serious Leisure Literature (SLI) that describe the process of intensifying the engagement in one’s hobby as a process that also works backwards (Liu et al., 2022). These categories are probably related to the hobby, semi-professional and professional categories in this study but for some (worker-pro/semi-professional) the link seems to be clearer than for others.

Nowadays, professional poker players often play partially with money from third-party investors (Holts & Surugiu, 2016). This is called “staking” and usually involves a fee that depends on the players skill and renown. This way, the poker player rents out his playing time for a certain income while minimizing his own risk. This seems sensible since playing poker professionally requires a huge investment in terms of personal resources in terms of time, money and mental energy (Hayano, 1977, p. 559f). The present study also integrates this aspect and also checks whether there are differences in any additional sources of income between hobby and (semi-)professional players. Professional poker players also acquire working routines that “resemble legitimate non-gambling work…” (Hayano, 1977, p. 559). Ongoing self-improvement is also an important aspect for the professional players (Hayano, 1977, p. 559).

The relevance of status for professional players has been mentioned before (Hayano, 1977, p. 559) and is integrated in one of Salaman’s determinants of occupational communities. Hayano claims that status is gained primarily by consistent winning at the tables, though there seems to be a generational conflict in professional poker regarding the way these winnings are achieved. While the older players claim that success is based on experience, the young attribute it to statistics and disciplined study (Hayano, 1977, p. 559f). Hing et al. (2015, p. 1806, 2016, p. 250) found that only a small minority of the overall player base considers themselves as professional (1.2%) or semi-professional (6.8%) and that even these people were likely to face gambling related social problems. Other studies show that pathological gambling is relatively rare, at least among online players. They also seem to be low in negative psychological traits and high in self-esteem (Biolcati et al., 2015). The low overall numbers of professional gamblers can be explained by at least two things. Players compete with each other and have to “beat” the rake on top of beating the other players in the long term to stay profitable. This establishes a natural boundary for the amount of (semi-)professional players in the system. Another reason for the small number of professional players is the fact that most players attempt to move up the limits during their poker career, in order to reach an average dollar amount where it becomes lucrative to partially or fully replace other sources of income with poker. Only a minority of players are successful in this attempt (Hayano, 1984, p. 133) similar to professional horse race gambling (Rosecrance, 1988, p. 234). Vertical mobility to other industries is also very unlikely, since the acquired skills from poker are very specific and rarely transferable to other occupations. The frequent drop-outs from this success pipeline also explain why studies show, that social relations among professional poker players seem to matter when it comes to money lending and mutual trust. Only the most successful and renowned players have access to the full range of “social options” (1977, S. 562). Some professional poker players even lie about their source of income amongst friends and family (Hayano, 1977, p. 562).

## Data

The present study includes data from 109 poker players from all active poker facilities in North-Rhine Westphalia, collected from 2022 to 2023 in the casinos in Aachen, Bad Oeynhausen and Hohensyburg (Dortmund) with the support of the local providers.^2^ Salaman (1974, p. 20) does not include occupational communities that depend on monopolist company in his framework. This is the case to a certain degree in the present study since the state of North-Rhine-Westphalia only has one licensed casino provider. However, many players travel to other states to participate in poker tournaments and also regularly play online. The pros and cons of this deviation will be discussed in the final section of this article. The survey was available for players at the cash-game tables on selected weekends and during a large multi-day tournament in Aachen (440€ buy-in). The survey was pretested by 12 players, recruited from various online poker forums. This pretest included a form for full-text feedback in case some important aspect was missing from the survey. For the final survey, the players were able to access the online survey through their smartphone via QR codes placed directly on the poker tables. The respondents could choose between a German and English version of the survey, but almost all players used the German form. To incentivize participation, 50€ were raffled off between all players per evening for the cash-game tables and 4*50€ per day for the first 2 days of the tournament.

The survey started with an item that screened for the location of the casino and an initial filter-question where participants indicated whether they consider themselves a hobbyist, semi-professional or professional. The most important group of items for checking whether (semi)professionals are indeed an occupational community are an individual’s willingness to professionalize their poker play, the societal view of poker players and stigmatization as well as the importance of social aspects in poker. For the purpose of easier interpretation and visualization, all variables from the attitudinal scales are dummy-coded from the original four-point Likert scales, so that the interpretation only considers agreement/disagreement. The questions on the poker-related ego-centric network of the player also help to answer the research question. The size of the overall network was gathered by asking the respondents to estimate the sum of all regular poker-related social contacts. Afterwards, the participant was asked to name up to five of their most important social contacts from poker. These name generator questions are typical hints in ego-centric network research to gather ego-alter ties (Crossley et al., 2015, p. 50). After this, the respondents indicated for each alter whether they were considered a “friend”, “mentor”, “colleague” or “competitor”. Multiple selections were allowed since an overlap of these categories seemed quite likely. This non-exclusivity of categories comes with some downsides since some ego-centric measures assume exclusivity (Crossley et al., 2015, p. 79; McCarty et al., 2019, p. 159). However, this was only a minor issue for the present study where the network data is used for descriptive analysis. The survey also contained items that considered poker-related playing behavior (limits, variants, frequency, stake, winnings/losses etc.), the respondent’s participation in other gambling activities in the last 12 months and items on the sociodemographic background. Table 1 shows the descriptive distribution of all variables in the study, separated by hobbyist and (semi-)professional players. A more detailed description of all variables in the study can be found in part one of the Appendix. Additional variables from the survey such as the distribution of play time between specific variants of poker as well as participation in other gambling related activities such as Lottery, sports betting or slot machine gambling as well as a detailed breakdown of some of the education and income variables are presented in part two of the Appendix.

**Table 1:** Descriptive statistics of all variables split by “Player category”

	N	Mean/prop	SD	min	max
**Hobby players**					
*Gambling behaviour*					
Location == Aachen	80	0.29		0	1
Location == Bad Oeynhausen	80	0.35		0	1
Location == Dortmund/Hohensyburg	80	0.36		0	1
Micro stakes	78	0.19		0	1
Low stakes	78	0.23		0	1
Medium stakes	78	0.44		0	1
High stakes	78	0.1		0	1
Super high stakes	78	0.04		0	1
Average play time	77	12.28	11.92	0.5	50
Win amount	51	4188.27	7228.79	0	45,000
Loss amount	22	2502.5	7807.93	0	37,000
Revenue	78	20.68		0	100
Online casino	77	24.94		0	100
Casino	77	51.82		0	100
Private games	77	23.25		0	100
Additional income	80	1.07		1	2
*Attitudinal variables*					
Job meaning	0	0		0	0
Professional ambition	77	0.16		0	1
Beat	61	0.34		0	1
Learnings	77	0.44		0	1
Poker view	74	0.5		0	1
Stigma	77	0.68		0	1
Stigma pro	77	0.66		0	1
Social relationship	76	0.67		0	1
Importance	78	0.72		0	1
*Network variables*					
Network size	76	24.3	43.08	0	250
Friends total	63	2.92	1.95	0	5
Mentors total	61	0.34	0.68	0	2
Colleagues total	58	1.36	1.75	0	5
Competitors total	55	0.64	1.25	0	5
*Demographics*					
University education	78	0.47		0	1
Fulltime employment or self-employed	79	0.78		0	1
Sex	77	0.88		0	1
Age	75	40.51	12.75	18	74
Migration	77	0.19		0	1
Migration parents	73	0.3		0	1
Monthly net income	71	3527.41	4176.17	0	30,000
**(Semi)professional players**					
*Gambling behaviour*					
Location == Aachen	29	0.45		0	1
Location == Bad Oeynhausen	29	0.34		0	1
Location == Dortmund/Hohensyburg	29	0.21		0	1
Micro stakes	29	0		0	0
Low stakes	29	0.14		0	1
Medium stakes	29	0.34		0	1
High stakes	29	0.48		0	1
Super high stakes	29	0.03		0	1
Average play time	29	28.45	19.73	4	100
Win amount	26	14,576.76	20,697.2	25	100,000
Loss amount	1	4500		4500	4500
Revenue	29	27.59		0	100
Online casino	29	25.17		0	95
Casino	29	65.17		5	100
Private games	29	9.66		0	70
Additional income	29	1.45		1	2
*Attitudinal variables*					
Job meaning	28	0.96		0	1
Professional ambition	0	0		0	0
Beat	27	0.59		0	1
Learnings	29	0.28		0	1
Poker view	29	0.59		0	1
Stigma	27	0.81		0	1
Stigma pro	29	0.76		0	1
Social relationship	29	0.72		0	1
Importance	29	0.83		0	1
*Network variables*					
Network size	29	22.86	30.61	1	150
Friends total	19	2.79	1.96	0	5
Mentors total	18	0.44	0.70	0	2
Colleagues total	18	1.17	1.34	0	4
Competitors total	17	0.94	1.43	0	5
*Demographics*					
University education	29	0.45		0	1
Fulltime employment or self-employed	29	0.83		0	1
Sex	29	0.97		0	1
Age	29	36.69	9.20	21	60
Migration	29	0.14		0	1
Migration parents	28	0.25		0	1
Monthly net income	27	3121.59	1652.55	900	8333

## Results

Before examining the hypotheses, I describe the sample of poker players in the study with regards to their gambling activity and demographic characteristics. In the sample, 73.4% people self-identified as hobby players, 24.8% as semi-professional and 1.8% as professional players. For most analyses, semi-professionals and professionals were grouped together by creating a new dummy variable. Table 1 presents the main results. It compares (semi-)professional players with hobby players on distinctive characteristics. For the purpose of many of the following analyses, the hobby players serve as a reference group to which the (semi-)professionals can be compared.

### Description of the Sample: Playing Frequency, Buy-Ins, Revenue, Online Poker

First, I will consider the average play time, the buy-ins (“stakes”) and the revenue from poker. (semi-)professional players spend much more time playing poker per week with a mean of 28.45 h compared to hobby players who only play 12.28 h per week on average. 51% of the (semi-)professionals participate primarily in games/tournaments with a buy-in above 301€ (“high stakes” or “super high stakes”) while only 14% of the hobby players play regularly at these levels. Participants indicated whether they won or lost money playing poker in the last 12 months. While the hobby players reported average winnings/losses in the past 12 months of 4188.27/2502.5€, the (semi-)professionals stated 14,576.76€/4500€. Multiplying these averages by the number of people in each group yields total amounts of wins/losses of 213,602/55,055€ for hobby players and 378,996/4500€ for the (semi)professional players. These numbers seem quite positively skewed. Possible reasons for this are discussed in the discussion section. For the purpose of this study, the exact number are less relevant than the group comparison. It is clear that (semi) professionals estimate their prior wins much greater than their prior losses and that the ratio is much more positive compared to the hobby players confirming the initial expectations. The buy-ins, the average play time and the revenue are a clear indication that the subjective self-categorization, i.e. the hobby/(semi-)professional categories directly relate to quantitative measures and all results confirm the basic assumptions about these variables as differentiating factors between hobby and professional players. Moreover, this confirm that players in the study perform a valid self-assessment regarding the category they belong to.

Based on prior research, I expect that (semi) professional players are more likely to use the additional opportunities that internet poker offers. The revenue variable shows that (semi-)professionals generate a higher percentage of their revenue from online poker compared to hobby players. The distribution of play time between live casinos, online casinos and private home games also shows that hobby players play more in a private environment, while (semi-)professionals prefer both online and live casinos, which is the primary playing environment for both groups. Based on previous research, I expect that (semi-)professional players have additional sources of income which are related to their poker playing. The variable “Additional income” combines multiple poker-related sources of income such as advertising deals or staking. We see that (Semi-)professionals generate income through these sources more frequently.

### Description of the Sample: Demographics

I will compare the hobby against the (semi-)professional players with regard to their demographic characteristics. To simplify the analysis of the educational variables, I summarize all categories above/below a Bachelor’s degree. Both groups have relatively similar levels of education, with 47.44% of the hobby players and 44.83% of the (semi-)professionals having at least a Bachelor’s degree. This shows that poker players are a very highly educated group compared to the German population as a whole, where only 18.5% have any form of university education.^3^

I also categorize occupational status in either group, comparing full-time employed & self-employed against all other categories. This provides us with an idea how the categories (hobby vs. (semi-)professional) relate to other occupational activities and how many of the players obtain a significant share of their income from poker. Hobby players are full-time or self-employed in 79% of cases compared to 83% of (semi-)professionals. However, the interpretation of this number is not necessarily straightforward for (semi-)professional players because it is unclear whether they regard poker as their occupation for the sake of this question. A closer look at the two professional players in the sample can be helpful here. One of the two professional players stated past year winnings of 100,000€, a monthly net income of 6000€ and self-employment—so poker is very likely his only source of income.^4^ The other person stated past year winnings of 24,000€ and a monthly net income of 3400€. This indicates that there might be an additional source of income involved; the person also stated additional incomes related to poker, so it is very likely that he makes all his net income from poker and related activities. This shows that the professional players in the sample obtain all of their income from poker. For the (semi-)professional players, we can assume that most of them have another major source of income aside from poker as indicated by the income values that are discussed in the next paragraph.

While the sample is dominated by male poker players (96 males vs. 10 females and one missing value), the ratio of males to females is much higher in the group of hobby players than in the group of (semi-)professionals. Hobby players are also about three years older on average and more likely to have a first- or second-generation migration background. The average monthly net income is 3527.41€ for hobby players and 3121.59€^5^ for (semi-)professionals. Even if both values might be slightly overestimated because of misreporting and the resulting data cleaning that is explained in the “Appendix” in the description of the variable “net income”, the income of both groups is high compared to the mean income in Germany.^6^

### Testing the Hypotheses

I start with H1a-H1c that check whether the components of occupational communities can be identified for the (semi) professional poker players in this study. H1a suggests that (semi-)professional players should treat the activity as an important part of their lives aside from the financial aspects. Of the (semi-)professional players 96% indicate that poker is clearly regarded as an important aspect of their lives aside from the aspect of making money (“Job meaning”). The variable “Importance” captures whether poker is important for the players beyond the financial aspect. This seems to clearly be the case for the majority of players, but is especially the case for (semi-)professionals (71.79% vs. 82.76%). This shows that poker is similar to other types of work in that regard. Poker seems to offers some intrinsic qualities to some people that are not necessarily obvious to all outside observers. From the variable “Social relationships” we see that contacts in poker are important for many of the players. This is slightly more often the case for the (semi-)professionals and shows that poker has a meaning for the players aside from the gameplay or the money. In summary these results clearly show that poker is an important element in the lives of the (semi-)professional players and confirm H1a.

H1b relates to the reference group as a component of occupational communities. I expect that (semi-)professional players consider other poker players as a reference group by defining poker-related social contacts as colleagues. The relatively large personal networks of frequent poker contacts (24.3 vs. 22.86) provide us an idea of the scope of these poker networks. Of the five closest social contacts, hobby players state less mentors and competitors, but they have more friends and colleagues than the (semi-)professional players. It makes sense that (semi) professional player name more alters as mentors or competitors since the hobby players might not seek self-improvement to the same degree and are probably not so concerned about winning/competition. The most important aspect for regarding others from the same occupation as a reference group are shared norms and values, which is inherent in colleague relationships. However, hobby players categorize more of their close contacts as “colleagues” than (semi-)professional players. Hobby players on average name 1.36 close social contacts as colleagues while (semi-)professionals consider 1.16 people as colleagues. However, this must not be a clear indication that (semi-)professionals do not share values and norms, but rather that it happens for all players. Therefore, the reference group might be more industry-based rather than based on occupational status. It might also be problematic that the present sample contains mostly (semi-)professionals, since the few fully professional players might share their own set of values and only relate to each other in terms of a reference group. Nevertheless, this is rather unlikely since the group is so small and it most likely needs a certain amount of people to speak of a true reference group. In summary, H1b can be confirmed even if the reference group is not exactly pinpointed.

In H1c I expect that (semi-)professional poker players consider other poker players as friends. This is clearly the case since “friends” are by far the largest category of the close social contacts in the ego-centric network of the players. However, hobby players in the study identify slightly more of the close poker-related contacts as friends (2.92 friends) but this is also the most prevalent group of contacts for the (semi)professionals by quite a significant margin (2.79 friends). H1c can therefore be confirmed.

Next, the three determinants for occupational communities are checked based on the data. In H2a, I expect that (semi-)professional players have a high level of involvement in poker in comparison to hobby players. The results on H1a already indicate that (semi-)professionals value the activity beyond the financial aspect and treat is as an important part of their lives in general. While this does already indicate some form of involvement, we have to take a look at the required individual skill which has been identified as one of the major indicators of involvement. The majority of hobby players do not think that the games became tougher/harder to beat in the last 24 months, but (semi-)professionals think differently in the majority of cases. The variable “Learnings” shows that hobby players think that it is generally easier to become a professional poker player than (semi-)professionals do. This shows that hobby players seriously underestimate the difficulties that (semi-)professional players have to overcome and the skill that is required for it. If the skill requirements were not as high, many hobby players would probably try to improve their game to the next level. However, the hobby players are rarely dedicated to do this. Only 15.58% of hobby players indicate an interest in becoming a professional poker player. This indicates a high level of involvement of the (semi-)professionals to achieve and maintain this status/level of play confirming H2a.

H2b states that (semi-)professional players are characterized by a marginal status or stratification situation. I argue that status is not only about peer-group comparisons but also about the societal view in general. Both groups of players are more or less indecisive whether their involvement in the game is viewed as something positive or negative. The (semi-)professionals are slightly less concerned in this regard since 59% of them see it as something positive while 50% for the hobby players think this way. Both the hobby as well as the (semi-)professionals have rather negative views when being asked more directly whether others think highly of professional and non-professional poker players. Out of the entire sample, 74 people agree to the statement “poker players are less appreciated in our society” while 30 people disagree. The (semi-)professionals agree with the statement much more often than the hobby players (68% vs. 81%). These number are very similar for the “Stigma pro” variable (73 vs. 33) so there seems to be no meaningful difference between poker players as an overall group and professional players specifically with regard to their societal image. Here, again, the (semi-)professionals are much skeptical about the overall societal image (66% vs. 76% agreement). In combination we can identify the (semi-)professional poker players as a marginal status group and confirm H2b. However, an outside perspective from people who do not play poker is missing from this study which lowers the impact of this hypothesis with regard to the research question. Since a marginal status or stratification situation is not a mandatory determinant for occupational groups, we can check whether the third determinant is present to reach a final conclusion.

H2c suggests that (semi-)professional players experience an inclusiveness of the work or the organizational situation. Since *pervasiveness* and *organizational embrace* are not relevant factors for (semi-)professional poker players, I focus on potential *restrictive factors*. We see that (semi-)professional players play much more hours per week (28.45 h) compared to hobby players (12.28 h). This restricts their ability to maintain social contacts outside of the poker setting and generally limits their options in terms of other activities because of the frequent work at night. We also see that (semi-)professional players play at higher stakes compared to the hobby players. This can be seen as another restrictive factor, since the high buy-ins drastically increase personal risk and the potential losses that can occur if the high luck factor that is also part of the game is not on the side of the players. In combination, these results show that the third determining factor, i.e. the inclusiveness of the work or organizational situation, can be assumed for (semi-)professional poker players. Hence, H2c can be confirmed by the data. Considering that all hypotheses have been confirmed, I conclude that (semi-)professional poker players form their own occupational community according to the criteria that Salaman identified.

## Discussion

Based on previous studies on the topic (Dufour et al., 2015; Hayano, 1977, 1984; Holts, 2017; Holts & Surugiu, 2016; McCormack & Griffiths, 2012; Radburn & Horsley, 2011), it was already quite clear that the term “profession” does not apply to gamblers regardless of their level of skill or involvement. Therefore, the present study examines the question whether (semi-)professional gamblers can be considered an occupational community according to sociological theory on work/leisure relationships by Salaman (1974) and ethnographic research on poker players (Hayano, 1977, 1982, 1984). We have seen that all common components of occupational communities can be found in the sample of (semi-)professional players. With regards to the three determinants of occupational communities some critical points have to be discussed.

The high level of involvement of the (semi-)professional players as the single mandatory determinant of occupational communities is a major finding of the study. The fact that hobby players rarely attempt to achieve professional status underlines this. Other studies also point out the high level of engagement of the professional poker players (Hayano, 1977, p. 559f) and this finding is in line with studies on professional horse race gambling (Rosecrance, 1988, p. 224) that ascribe a high level of commitment, discipline and effort to the professional gamblers.

Also in line with previous studies, the results indicate that (semi-)professional players are indeed a deviant group (Hayano, 1984; Salaman, 1974, p. 14f) or situated at the edge between deviant and legitimate (Radburn & Horsley, 2011, p. 30). It should be noted that this does not seem to be the case for all professional gambling. For professional horse race gamblers, this is not the case since “professionals did not consider their careers as deviant, nor did they believe that the larger society regarded their activities as abnormal or reprehensible” (Rosecrance, 1988, p. 227). Unfortunately, a valid outside perspective on poker is missing from the current study so it cannot be finally answered, whether the fact that the second (optional) determinant can be confirmed is really a meaningful indicator to categorize (semi-)professional gamblers as an occupational community.

However, the analysis of the third determinant leads to a clear overall picture showing that the (semi-)professional poker players in the study form an occupational community according to Salaman’s criteria. Inclusive factors are clearly present for the (semi-)professional poker players. While *pervasiveness* or *organizational embrace* play no role in (semi-)professional poker, the activity is characterized by significant *restrictive factors*. The much longer periods that (semi-)professionals spend in the casinos compared to hobby players restrict their ability to maintain social contacts outside of the poker setting and generally limits their options to participate in other activities because of the frequent work at night. They also play for larger amounts of money most of the time which increases the personal risk. This might be a serious cause of stress and restrict the (semi-)professional players overall well-being during periods of losses.

This study is not only relevant as another case study on occupational communities but also because it shows that Salaman’s concept encompasses even such communities as (semi-)professional poker players, who have been disregarded by prior research. This also means that the theoretical tools from his framework could be used to study other less recognized groups of people who obtain a significant share of their monthly income from activities that are not yet recognized as work. Thereby, this study is also a first step to breach the gap between activities that are commonly discussed under the label of the serious leisure literature (SLI) and those that are considered occupational or professional. Moreover, it might be a starting point for a more in-depths analysis of the challenges that this community faces with regard to access to social welfare benefits and possible job opportunities in other sectors for those that have to give up the occupation for various reasons.

We have seen that more than a quarter of the sample in the study identified as semi-professional or professional. This is a large number in comparison to prior studies. In the largest study of professional poker players to date (Hing et al., 2015, p. 1806, 2016, p. 250) very few of the players from Australia considered themselves as professional or semi-professional. This discrepancy can probably be explained by the different sampling frame. While this study was conducted in a real casino environment, the players in other studies were recruited from online advertisements.

It is also interesting that many (semi-)professional players in the study spend a significant share or their playing time online and also obtain a sizable share of their poker-related income from it. However, it can be questioned whether this will remain a viable practice in the future. Recently, strong AI programs were able to beat top players. At first only in heads-up (1v1) games (Brown & Sandholm, 2018) but the latest versions are even successful in multiplayer games (Brown & Sandholm, 2019).

The gambling industry is far too large and diversified to include every aspect of professional gambling. Instead, this study focused on poker since it is the game of choice for many professional gamblers (see Thorp, 2018 for an overview of other possible strategies). The merging of the two categories, semi-professional and professional poker players, resembles a more continuous approach to the analysis of professions and occupation, where the boundaries of each step in the professional hierarchy are more or less fluid (Volti, 2012, p. 156). While this provides the opportunity to analyze and compare larger samples of poker players, the obvious downside of this approach is the fact that clear-cut statements about specific individuals or subgroups are harder to extract. However, the primary goal of the present study was to uncover general occupational characteristics of (semi-)professional poker players as a community. With a sample full of professional poker players, the findings would be even less ambiguous, since semi-professional players are somewhere in-between both ends of the professionalization hierarchy indicating a good robustness of the analyses.

While the study is as encompassing as possible in the sense that it includes all casinos that provide poker in the German state North-Rhine-Westphalia, this also means that the dataset is only based on one monopolistic provider. This has to be mentioned since Salaman (1974, p. 20) related model of occupational communities only to those who form naturally and are not dependent on one local provider. However, he already agreed that some occupational would have formed regardless of it (Salaman, 1974, p. 21) and I would argue this is the case for (semi-)professional poker. It should not make any difference for the formation of the occupational group whether the casinos are operated by a monopolistic provider or many small ones, since they are not directly employers for the (semi-)professional poker players. In summary, the restriction of the framework does not diminish the value of the present analysis but on the contrary extends the theoretical debate to this rather special case, showing how widely applicable the framework is.

Another point of criticism could be the fact the study lacks any psychometric scales that could have explained whether differences in personality might partially explain why some people try to professionalize their own poker game while others have no intention in doing so. Unfortunately, the length of the survey was already at the limit of what the players consider too burdensome. This is also the reason why the study does not include any qualitative open-ended questions that might have helped to get an even better understanding of some of the occupational differences between more and less professional players. Future research should try to integrate some of these missing elements and replicate the findings for other fields of professional gambling, such as sports betting markets or highly speculative trading of financial products, which has already been linked to professional gambling (Rosecrance, 1988, p. 233f; Weidner, 2022, p. 4).

## Data Availability

The full dataset from the study can be obtained from the author upon personal request. This also includes the variables from the Appendix that have not been used for the analysis in the article itself.

## Appendix: Part 1: Description of All Variables

### Gambling Behavior Variables

#### Player Category

This variable captures the degree to which a player thinks of himself as a poker professional. The item was formulated: “Which category describes your current poker activity the best?” with the possible answers “Poker is my hobby”, “I play semi-professionally” and “I play professionally”.

#### Location

Participants selected the location of the casino where they participated in the survey.

#### Limits

The respondents were asked for the usual stakes they participate in. The scale consisted of the options micro (0–25€), low (26–100€), medium (101–300€), high (301–1000€) and super high (more than 1001€).

#### Average Playtime

This item asked for an estimate of the average weekly time spent on any variant of poker as a numerical input.

#### Win/Loss Amount

Respondents were asked to indicate whether they won or lost money playing poker in the last 12 months. The available options were “Yes” and “No”. Based on this initial filter question participants were asked to indicate the amount they won/lost during the last 12 months. One value of − 9000 from the variable “WinAmount” was recoded as a missing value because this was most likely a thinking or typing error from the participant.

#### Revenue

This variable captures the percent of revenue from online poker in comparison to terrestrial and private poker. This value helps to separate those who gain a primary share of the revenue from online play from those who make it in a live environment. Prior studies also indicate that both groups might be different in terms of their approach to the game and personal characteristics.

#### Playing Environment

To capture a player’s preference for different playing environments participants were asked to distribute 100% of their usual playing time between the three categories “online casino”, “real casino” and “home games”.

#### Second Income

This variable has been coded as a dummy variable from a range of questions on additional incomes that relate to poker but do not result from the activity itself. Respondents were asked to select each applicable option out of staking (lending money), streaming poker on the internet, poker coachings, advertising deals (not including typical rakeback deals). Since most of these options were selected relatively rarely, the final variable is coded as a dummy variable that separates between people that do or do not have one of these additional sources of income.

#### Playing Forms

The respondents were asked for the specific forms of poker they participate in by distributing a total of 100% of their usual play time across seven categories that range from large tournaments (MTTs) to heads-up games (1v1) and everything in between. The results of these variables are displayed in part two of the “Appendix”.

#### Playing Types

The respondents were asked to distribute 100% of their usual playing time across four established variants of poker with an additional fifth category “others” to allow for the option to assign playing time to less common variants. The results of these variables are displayed in part two of the “Appendix”.

#### Other Gambling Activities

These question where added to get an impression of the various other forms of gambling that hobby and (semi)professional players might engage in. The item itself was coded as a matrix question that captures all common forms of gambling from sports-betting to purchasing high risk financial products on a four-point scale consisting of the options “none”, “rarely”, “occasionally” and “frequently”. Since most participants answered the questionnaire on a mobile device, the matrix question converts to single drop-down menus for every category separately on these devices. The results of these variables are displayed in part two of the “Appendix”.

### Attitudinal Variables

These variables capture various personal attitudinal aspects regarding poker. The first two variables have been filtered based on the initial group variable, while all others relate to the entire sample. Unless otherwise stated, items in this category use a four-point Likert scale that ranges from “fully agree” to “fully disagree” but have been recoded as binary variable (agree/disagree) for an easier visualization and analysis.

#### Job Meaning

Semi-professional and professional players were asked “What do think about this statement? Poker is more than just a job.”

#### Professional Ambition

Hobbyists were asked: “Do you strive to become a professional poker player in the future?”.

#### Beat

This variable captures the degree to which the perceived overall level of difficulty increased in the last 24 months. The specific wording was: “Do you feel that games became harder to “beat” in the last 24 months?” Since a direct translation was not sensible, the German translation is formulated slightly differently but conveys a similar meaning. This item uses the four-point Likert-scale mentioned above but also includes the additional option “don’t know”.

#### Learnings

Since prior studies indicate that professional poker players are a very small and highly talented and committed group, the survey included a measure that captures whether players feel that success in the game is relatively achievable. The item was phrased: “Do you think most people can become a professional poker player when they spend enough time on learning the game?”. answers were restricted to “yes” and “no” to force a clear tendency.

#### Poker View

This item was added to the survey in order to get an idea of the general image of the poker player in our society. The item reads: “Do you feel that other people regard your poker play as something positive or negative?” The possible responses ranged from “very positive” to “very negative” on a four-point scale.

#### Stigma

This variable captures whether poker players are regarded as a stigmatized group in our society. For clarity, the item reads “poker players are less appreciated in our society”.

#### Stigma Pro

This variable is similar to the previous except that the wording was changed to “professional poker players” to check whether there are any meaningful differences.

#### Social Relationship

The item asks whether social relationships to other poker players are important to the participant.

#### Importance

This variable captures whether a person plays poker primarily because of the possible financial gains or not. The corresponding item in the survey was: “Poker has a meaning for me far beyond the financial”.

### Network Measures

#### Network Size

Participants were asked to indicate the overall amount of frequent poker-related social contacts they have as a numerical value. This is not related to the ego-network (next variable) but a global measure of estimated network size.

#### Friends/Mentors/Colleagues/Competitors Total

This ego-centric network data was collected in a two-step process. The survey included a name generator question where participants entered up to 5 names of their closest poker-related social contacts.^7^ In a follow-up question, the names of these alters were presented again and the respondents indicated whether each is considered a friend, mentor, colleague or competitor. Multiple selection was allowed because alters might reasonably fall into more than one of these categories.

### Demographic Variables

#### Education

Participants were asked for their highest educational qualification according to the International Standard Classification of Education (ISCED). The main article only contains a dummy variable that separates between university and non-university education. The results of all educational variables are displayed in part two of the “Appendix”.

#### Occupational Status

Participants selected their occupational status out of unemployed, minor employment, in training, retirement, self-employed, part-time employment and full-time employment. The original survey also contained additional information for some of the categories to help with the selection. The main article only contains a dummy variable that considers whether a person is full-time or self-employed. The results of all educational variables are displayed in part two of the “Appendix”.

#### Sex

The item in the survey reads “are you…”, the available responses for this item included the options “male”, “female” and “no answer”.

#### Age

The variable entails the current age of the respondents as a numerical value.

#### Migration Background

Since gambling preference and migration have been linked in previous studies and many young people in Germany are second-generation immigrants, a measure for personal migration as well as parent migration is included. The items asked whether the person/parent was born in a different country than Germany.

#### Monthly Net Income

The variable includes the personal monthly net income as a numeric value. In case the corresponding item was skipped in the survey, it was presented again as a categorical list with 16 categories from < 500€ to > 20.000€ and anonymity of the data was assured again. The option “no answer” was also included. Since only two respondents answered the categorical question, all responses are recoded into a new variable that is presented here called “net income”. A total of six values between 1 and 100 have been recoded as missing, since these seemed to be unrealistic values for full-time employment.

## Appendix Part 2: Descriptive Results for Additional Variables that have been Omitted from the Analysis

See Table

**Table 2:** Descriptive statistics of the omitted variables split by player “Player category”

	N	Mean/prop	SD	min	max
**Hobby players**					
*Playing forms*					
Fullring cash	78	46.03		0	100
Shorthanded cash	78	4		0	80
Headsup	78	0.96		0	50
MTT	78	30.29		0	100
MTT SNG	78	5.6		0	100
SNG	78	3.09		0	100
UTH	78	8.99		0	100
Other	78	1.04		0	50
*Poker types*					
No limit hold’em	78	80.33		0	100
Limit hold’em	78	5.77		0	100
PLO	78	10.41		0	80
Others	78	2.4		0	50
Mixed	78	1.09		0	60
*Other gambling activities*					
Blackjack	71	0.32		0	1
Roulette	72	0.49		0	1
Slots casino	73	0.32		0	1
Horse betting	70	0.03		0	1
Sports betting	68	0.41		0	1
Lotteries	70	0.53		0	1
Bingo	67	0.13		0	1
Slots amusement hall	71	0.21		0	1
Financial	72	0.4		0	1
Loot boxes	69	0.03		0	1
Scratch cards	66	0.18		0	1
*Education*					
Lower secondary education	78	0.05		0	1
Upper secondary education	78	0.09		0	1
Post-secondary non-tertiary education	78	0.06		0	1
Short cycle tertiary education	78	0.32		0	1
Bachelor level	78	0.33		0	1
Master level	78	0.13		0	1
Doctoral level	78	0.01		0	1
*Employment status*					
Unemployed	79	0		0	0
Minor employment	79	0		0	0
In training	79	0.08		0	1
Retirement	79	0.05		0	1
Self-employed	79	0.22		0	1
Part-time employment	79	0.09		0	1
Full-time employment	79	0.57		0	1
**(Semi)professional players**					
*Playing forms*					
Fullring cash	29	65.86		0	100
Shorthanded cash	29	5.17		0	50
Headsup	29	0.52		0	5
MTT	29	26.9		0	85
MTT SNG	29	0.52		0	5
SNG	29	0.17		0	5
UTH	29	0.52		0	15
Other	29	0.34		0	10
*Playing types*					
No limit hold’em	29	85.83		0	100
Limit hold’em	29	4.14		0	100
PLO	29	6.59		0	40
Others	29	1.03		0	30
Mixed	29	2.41		0	70
*Other gambling activites*					
Blackjack	25	0.24		0	1
Roulette	26	0.38		0	1
Slots casino	26	0.42		0	1
Horse betting	26	0.04		0	1
Sports betting	26	0.38		0	1
Lotteries	26	0.23		0	1
Bingo	25	0.04		0	1
Slots amusement hall	25	0.12		0	1
Financial	25	0.4		0	1
Loot boxes	25	0.04		0	1
Scratch cards	25	0.04		0	1
*Education*					
Lower secondary education	29	0.03		0	1
Upper secondary education	29	0.17		0	1
Post-secondary non-tertiary education	29	0.03		0	1
Short cycle tertiary education	29	0.31		0	1
Bachelor level	29	0.28		0	1
Master level	29	0.14		0	1
Doctoral level	29	0.03		0	1
*Employment status*					
Unemployed	29	0.03		0	1
Minor employment	29	0.03		0	1
In training	29	0.03		0	1
Retirement	29	0		0	0
Self-employed	29	0.21		0	1
Part-time employment	29	0.07		0	1
Full-time employment	29	0.62		0	1

^2^.

## Footnotes

1 The other criteria are: (1) general, systematic knowledge; (2) authority over clients; (3) community rather than self-interest; (4) self-control rather than outside control; (5) recognition by the community and in law that the occupation is a profession.

2 Merkur Spielbanken which is a part of the Gauselmann GmbH, one of the largest certified Casino providers in Germany, operating all state-licensed facilities in North Rhine-Westphalia and Saxony-Anhalt. The original aim of the research project was to provide a quantitative analysis of professionalization in poker by identifying key variables that distinguish professional poker players from hobby players. However, during the field phase of the study, it became apparent that the population of poker players in North Rhine-Westphalia was smaller than expected and that the response rates were rather moderate despite significant monetary incentives. Due to the lack of observations required for this large quantitative analysis, the research question has been adjusted to focus only on occupational group membership. All descriptive results of the study that are not relevant for this analysis are still shown in the “Appendix” to share these potentially valuable data with other researchers.

3 See: https://www.destatis.de/DE/Themen/Gesellschaft-Umwelt/Bildung-Forschung-Kultur/Bildungsstand/Tabellen/bildungsabschluss.html for an overview of the education data from the German federal statistics office.

4 Whether these winnings are gross values and taxes should be deducted for professional players remains a somewhat open question.

5 Both values might be slightly overestimated because of misreporting and the resulting data cleaning that is explained in the Appendix in the description of the variable “net income”.

6 See: https://www.destatis.de/EN/Themes/Labour/Earnings/Earnings-Earnings-Differences/Tables/quaterly-earnings.html for an overview of gross earnings in the Agricultural, industry and service sector from the German federal statistics office.
